# Biodiversity indices and Random Forests reveal the potential for striped skunk (*Mephitis mephitis*) fecal microbial communities to function as a biomarker for oral rabies vaccination

**DOI:** 10.1371/journal.pone.0285852

**Published:** 2023-08-22

**Authors:** Matthew W. Hopken, Darby Gilfillan, Amy T. Gilbert, Antoinette J. Piaggio, Mikaela Samsel Hilton, James Pierce, Bruce Kimball, Zaid Abdo

**Affiliations:** 1 United States Department of Agriculture, Animal and Plant Health Inspection Service, Wildlife Services, National Wildlife Research Center, Fort Collins, Colorado, United States of America; 2 Department of Microbiology, Immunology, and Pathology, Colorado State University, Fort Collins, Colorado, United States of America; 3 Monell Chemical Senses Center, Philadelphia, Pennsylvania, United States of America; Institute of Biological Sciences, University of the Philippines, PHILIPPINES

## Abstract

Wildlife disease surveillance and monitoring poses unique challenges when assessing rates of population vaccination, immunity, or infection prevalence. Non-invasively detected biomarkers can help reduce risk to both animal and field personnel during wildlife disease management activities. In this study, we investigated the utility of fecal microbiome data collected from captive striped skunks (*Mephitis mephitis*) in predicting rabies virus vaccination and infection status. We sequenced the hypervariable region 4 (V4) of the bacterial 16S gene and estimated alpha and beta diversity across timepoints in three groups of skunks: vaccination then rabies virus infection, sham vaccination then rabies virus infection, and rabies virus infected without vaccination. Alpha diversity did not differ among treatment groups but beta diversity between treatments was statistically significant. The phyla Firmicutes and Proteobacteria were dominant among all samples. Using Random Forests, we identified operational taxonomic units (OTUs) that greatly influenced classification of fecal samples into treatment groups. Each of these OTUs was correlated with fecal volatile organic compounds detected from the samples for companion treatment groups in another study. This research is the first to highlight striped skunk microbiome biodiversity as a vaccination biomarker which pushes the frontier on alternative methods for surveillance and monitoring of vaccination and disease in wildlife populations.

## Introduction

Most of our knowledge of non-human animal microbiomes comes from studies on laboratory animals or livestock, with a handful of studies on wildlife [1, 2]. However, there is now greater recognition of the role wildlife-specific microbiomes play in understanding and managing wildlife and associated pathogens [3, 4]. The microbiome is a crucial part of the mammalian immune system which can help determine infection outcomes by preventing the establishment of pathogens or facilitating immune responses [5]. Pathogens and other immune system triggers, such as vaccination, can induce concomitant responses in microbiome community structure. In some cases, immune system activation has predictable outcomes on the microbiome which has led some to suggest that alterations to taxonomic diversity can be used as a host biomarker of pathogen exposure, vaccination, and immune response [e.g., 6–9].

In wildlife disease management, one challenge is determining the extent of infection, vaccination, and immunity in natural populations. Biomarkers can inform monitoring and surveillance by providing vaccination estimates and non-invasive biomarkers could greatly ease the burden on agencies involved with animal trapping for disease surveillance and management. Biomarkers can include volatile organic compounds (VOCs) excreted by wildlife in urine, feces, and breath, and have been used to detect pathogen infections or vaccination status [e.g., 10–13]. Given that VOCs are often byproducts of microbial physiology, one can expect that changes in microbial communities may be correlated with changes in VOC profiles. Thus, if VOCs can be utilized as biomarkers of pathogen infection and vaccination, then the underlying host microbiome may also serve this purpose.

Shifts in microbial communities of wild mammals in response to environmental influences have been demonstrated for stressors such as habitat fragmentation, pollution, disease, and hibernation [3, 4, 14, 15]. While researchers have been able to detect shifts in microbiome communities, one question often remains: can certain microbial communities predict the presence or exposure to a immunological stressor, such as a pathogen or vaccine? Abdo, LeCureux [7] identified bacterial operational taxonomic units (OTUs) in lab mice that were associated with treatment groups in a study on a probiotic oral vaccine vector for human immunodeficiency virus (HIV). In two similar studies, Zhang, Wu [6] also found bacterial taxa that were associated with rabies virus infection and oral rabies vaccination in lab mice, and Shi, Zou [16] found segmented filamentous bacteria (*Candidatus arthromitus*) helps prevent rotavirus infection and alleviates infection-related symptoms in a murine model.

While little is known about the use of microbiomes as biomarkers, they hold great potential for utility in wildlife disease surveillance. One pertinent example of using the microbiome to detect pathogen infection was the identification of cloacal bacterial OTUs that were associated with influenza A virus infection status in wild birds [17]. Collecting fecal microbiome samples from wildlife is considered a non-invasive sampling technique that reduces the burden and risk to the animal and field personnel while allowing for collection of robust sample sizes [18]. Further, individual genetic identification can be obtained from fecal samples, and when used in conjunction with mark-recapture approaches, the population proportion of animals that are infected or vaccinated could be estimated.

Rabies lyssavirus (i.e., rabies virus) is a highly lethal zoonotic pathogen that can infect all mammal species. Rabies virus predominantly occurs in Chiropteran and Carnivoran wildlife reservoirs, with co-evolved variants that circulate within a single species with occasional spillover events to other mammals [19–21]. On the North American continent, rabies virus lineages of bat-origin and domestic dog-origin (*Canis lupus familiaris*) circulate in wild mesocarnivore species [21]. Within these clades there are subclades of variants that are found primarily in specific species [22, 23]. In the United States (U.S.), the United States Department of Agriculture (USDA), along with other Federal, state, and local agencies, work to mitigate the risks posed to humans, domestic animals, livestock and wildlife from rabies virus, by preventing the spread and local elimination of the raccoon (*Procyon lotor*) variant of rabies virus in the eastern U.S., and maintaining a canine rabies virus free status, through a program of oral vaccination and enhanced surveillance [24, 25]. In the central and western U.S., striped skunks (*Mephitis mephitis*) are one of the principal wildlife reservoirs for rabies virus consisting of three variants: northcentral skunk, southcentral skunk, and California skunk [23]. Striped skunks have also been recognized as the most frequent spillover recipient of raccoon variant rabies virus and are a key secondary target of control efforts in the eastern U.S. [26, 27].

The USDA currently conducts enhanced surveillance of rabies virus in mesocarnivores to track changes in epizootiology and to evaluate the effectiveness of vaccination programs for control and elimination [28]. Sampling requires trapping and handling of animals, which are potentially rabid. Field staff may also check for oral vaccine bait consumption by removing the premolar of captured raccoons; where vaccine-baits contain tetracycline which incorporates into the teeth functioning as a biomarker [29]. A non-invasive surveillance and monitoring tool could be a useful alternative to current strategies by alleviating the need to capture or handle animals to remove brain tissues or teeth respectively. Two studies have demonstrated that fecal samples may be an effective way to detect rabies virus infection and vaccination. Kimball, Volker [12] determined that certain fecal volatile organic compounds (VOCs) collected from captive striped skunks were correlated with rabies virus binding antibody response to oral rabies vaccination. Zhang, Wu [6] demonstrated that rabies virus neutralizing antibody (RVNA) response to vaccination was degraded following antibiotic treatment of lab mice and that certain bacterial taxa detected in fecal samples were positively or negatively correlated with high and low RVNA titers. In this follow up study to Kimball, Volker [12] we collected fecal samples from captive striped skunks used in oral rabies vaccine efficacy trials, to evaluate whether vaccination and/or rabies virus infection alters the fecal microbiome and thus could be used as an indicator of vaccination status.

## Methods

### Ethics statement

Animal use and procedures described were consistent with the guidelines of the USDA National Wildlife Research Center (NWRC) Institutional Animal Care and Use Committee (protocol QA-2258) and import and housing of skunks were permitted under Colorado Parks and Wildlife 13TR2056A1, 14TR2056, and 15TR2143.

### Vaccine trials and fecal collection

The animal handling and experimental treatments are described in detail in Gilbert, Johnson [30] and Kimball, Volker [12] but we provide a brief summary of vaccination, rabies virus infection, and fecal collection. We included samples from 22 animals in this study which was a subset of the 35 in Gilbert, Johnson [30] and included all animals from Kimball, Volker [12]. The vaccinated individuals were randomly assigned to one of four groups. Three of the groups received three different doses of Ontario Rabies Vaccine, ONRAB (Artemis Technologies, Inc., Guelph, Ontario, Canada) diluted in minimal essential media (MEM) supplemented with 5% fetal bovine serum (10^10.2^, 10^9.8^, or 10^9.3^ median tissue culture infective doses (TCID_50_; all combined into group Rvax for this study)). Ten skunks were given the oral rabies vaccine (group Rvax), five animals were given sham vaccine orally (MEM only; Group SHvax), and seven skunks were unvaccinated and challenged with rabies virus (no vaccine; group Rinf). The vaccine or sham treatments were administered to individual skunks by direct instillation into the oral cavity under light anesthesia with isoflurane gas [30]. On day 335 post-vaccination skunks were challenged with a New York City dog variant of rabies virus (92-5A) as described in Gilbert, Johnson [30].

Control skunks (group Rinf) were inoculated with 92-5A rabies virus as described in [30]. These individuals were randomly sorted into two groups that received different doses of rabies virus IM in bilateral masseter muscles. One group received 10^5.9^ MICLD_50_, while the other received 10^6.9^ MICLD_50_ in total volume of 1.0 mL. Rabies virus infection was definitively diagnosed by direct fluorescent antibody assay (DFA) at the Colorado State University Diagnostic Laboratory from collected brainstem and cerebellar tissues [30].

Skunks were individually housed and the day before fecal collection all feces was removed from the pens.Fecal samples that appeared within the last 24 hours were collected from the pen floor, cage catch pan, or den box and the freshest of those fecal samples were preferentially collected [12]. During the vaccination trial, fecal samples were collected at days zero, 29, 60, and 335 post-vaccine (pv). Fecal samples were also collected after the rabies virus challenge on day 14 post-infection, unless the animal was euthanized due to illness before the full 14 days. Three of five animals in the Rinf group, one of three animals given SHvax group, and none of the Rvax animals were euthanized before 14 days post infection. Approximately 2-5g of feces were directly deposited into whirl-pak specimen bag. Samples were stored on ice packs for up to 6 hrs after collection and then stored in -80°C.

### Sample processing and sequencing

Samples were extracted using Dneasy® PowerSoil® Kit (Qiagen, Hilden, Germany) according to manufacture directions. We used this extraction kit on all samples to prevent introducing biases from cross-kit comparisons [31]. DNA extracts were stored in -80°C until they could be preppared for sequencing. For each sequencing run we included a commercially available mock community of bacterial DNA, processed through the same library preparation as the samples, for monitoring error and quality control (ZymoBIOMICS microbial community DNA standard, Zymo Research, CA, USA cat. D6306).

To evaluate the fecal microbiome we used a two-step amplicon sequencing approach similar to Galan, Pons [32]. We first amplified the hypervariable region 4 (V4) of the 16S rRNA gene using a multiplex of primers that were based on 515F and 806R primers [33, 34]. We modified the primers to contain different heterogeneity spacers on the 5’ end between partial sequencing adaptors and the amplification primer sequences (S1 Table). All PCR steps were amplified using AccuPrime™ Pfx DNA Polymerase (Invitrogen, Carlsbad, California, USA). The PCR mix contained 25μL of AccuPrime™, 1μL at 10μM for each primer mix, and 5μL of template DNA. The cycling conditions were an initial melting phase at 95°C for 5 min, then 25 cycles of 95°C for 30 sec, 50°C for 60 sec, and 68°C for 98 sec, and a final extension at 68°C for 5 min. To eliminate contamination by primer dimers from 16S amplification, which can decrease sequencing quality, samples were purified using Mag-Bind® TotalPure NGS (Omega Bio-tek, Inc., Norcross, Georgia, USA) beads using a 0.8 volume of beads and eluted in 50μL to dilute samples to improve the second PCR amplification.

The second PCR added sequencing adaptors and dual indices to the purified amplicons (S1 Table). This reaction included a PCR mix containing 25μL of AccuPrime™, 1μL at 10μM of each primer, and 3μL of purified template. The cycling conditions were as follows, 95°C for 5 min, then 8 cycles of 95°C for 30 sec, 50°C for 60 sec, and 68°C for 98 sec, and a final extension at 68°C for 5 min.

As the final purification step, the samples were normalized on SequalPrep™ Normalization Plate Kit (Applied Biosystems, Foster City, California, USA) according to manufacturer’s directions. A subset of samples was checked for normalization of concentration using Qubit™ dsDNA HS Assay Kit (Invitrogen, Carlsbad, California, USA). Samples were then pooled in equal volumes and preppared for paired-end sequencing on an Illumina MiSeq System; we used the 500-cycle MiSeq Reagent Kit v2 (Illumina, San Diego, California, USA).

### Bioinformatics

Illumina sequencing fastq files were demultiplexed and read quality was evaluated using FastQC v.0.11.5 [35]. Reads were then quality-filtered using trimmomatic v.36 set at a sliding window of four, optimum PHRED quality score of 25, and a minimum read length of 150 [36]. Files containing zero reads or labeled as unidentified were removed from further data processing to prevent issues in downstream analyses. Taxonomic classification of samples was performed using mothur v.1.44.0 [37] implementing the PE-de-novo-processing script available at the Abdo-Lab/Microbiome-Analysis-Scripts GitHub [37]. Chimera identification within mothur was completed using vsearch v.2.13.3 and silva v.132 as the reference database [38–40].

### Statistical analyses

Statistical analyses were completed in R v4.1.3 and RStudio v2022.2.1.461 [41] which included removal of OTUs that were present in extraction blanks (EB) and negative controls. The following statistical analyses were conducted on grouping the sampling timepoints into three groups, Rvax = vaccine treatment, SHvax = sham vaccine, and Rinf = rabies virus infection only. The analyses were conducted on a randomized, repeated measures study design within treatment groups that were self-controlled. Each subgroup included samples collected before any treatment to determine the baseline skunk microbiome within groups (Rvax-start, SHvax-start, Rinf-start), two treatment subgroups (Rvax, sham vaccine SHvax) and three post rabies virus infection subgroups (Rvax-infect, SHvax-infect, Rinf-infect; Table 1, S2 Table). Normality of sequencing depth, number of datapoints, number of OTUs, and number genera per animal was evaluated with a Shapiro-wilks test. We calculated alpha diversity indices, Shannon diversity, Chao1, inverse Simpson (InvSimpson), and rarified species richness, using vegan and phyloseq package v.1.38.0 [42]. We used a rarefaction curve produced by the vegan package v2.6–2 [43] to evaluate the relationship between host-species richness and read depth per sample. Alpha diversity index confirmation to normality was tested using the Shapiro-Wilks test. For indices that were normally distributed, we tested for significant differences among treatment subgroups using a generalized linear model (GLM) and analysis of variance (ANOVA). For indices that deviated from normality we evaluated significance among treatment subgroups using the Kruskal-Wallis rank sum test. Barplots of normalized relative abundance of bacterial genera per treatment subgroup were created using ggplot2 [44] with a minimum relative abundance of 0.01. Venn diagrams of shared OTUs among treatment subgroups were generated with ggvenn v0.1.9 [45] with each group, Rvax, SHvax, and Rinf, shown separately. Visualization of data trends and clustering of microbial community structure per sampling time point (see experimental design in Table 1) was conducted using nonmetric Multidimensional Scaling (NMDS) [46] on OTUs and using Bray-Curtis dissimilarity [46] with OTU data normalized using Cumulative Sum Scaling (CSS) [47]. Significance of beta diversity was assessed among subgroups within each treatment group separately, Rvax, SHvax, and Rinf, using PERMANOVA [48] with 1000 permutations. For significant PERMANOVA we tested for the influence of lack of homogeneity of variance using a beta dispersion test with 1000 permutations. Relative abundance of CSS normalized OTUs was estimated using the phylosmith v1.0.6 package [49]. The Spearman rank correlation coefficient was used to evaluate correlation between OTU relative abundance in the Rvax (vaccination time points only) and fecal VOC peak data from Kimball, Volker [12] to determine if VOC concentration is correlated to specific bacterial taxa in vaccinated animals.

**Table 1:** Summary data for each treatment group and subgroup of skunk microbiome data which includes the number (#) of animals in each group, the number (#) of samples datapoints in each subgroup the mean sequencing depth and range, and the number operational taxonomic units (OTUs) and genera recovered from the sequence data. Also presented are the mean (range) of alpha diversity metrics estimated for each treatment subgroup.

Group	Animals	Subgroup	Datapoints	Mean depth (range)	OTUs	Genera	Richness	Chao-1	Shannon	InvSimpson
Vaccination	7	Rvax-start	7	108965 (35965–153267)	99	35	19.83 (12.3–31.04)	35.57 (21–63)	1.24 (0.95–1.53)	2.31 (1.87–3.2)
		Rvax	18	56418 (6078–169191)	108	36	17.12 (7.53–36.94)	24.71 (10–52)	1.48 (0.36–2.68)	3.49 (1.15–9.14)
		Rvax-infect	6	49353 (32172–62471)	50	19	18.55 (12.06–25.54)	24.83 (16–35)	1.42 (0.44–2.2)	3.58 (1.17–7.01)
Sham vaccine	3	SHvax-start	3	27319 (1013–48393)	32	18	16.69 (13–21.5)	21.33 (13–29)	1.53 (1.35–1.8)	3.1 (2.57–3.86)
		SHvax	6	26404 (1515–84742)	44	20	11.53 (6.99–22.82)	15.33 (7–38)	1.48 (0.46–2.21)	3.89 (1.27–7.28)
		SHvax-infect	3	22585 (3185–33274)	44	20	19.85 (9.17–31.75)	25.83 (11.5–41)	1.74 (1.61–1.87)	3.54 (3.09–4.15)
Infection only	5	Rinf-start	5	57782 (24475–79920)	95	42	26.77 (15.17–39.86)	39.8 (23–71)	2.03 (1.57–2.49)	5.08 (3.17–7.46)
		Rinf-infect	5	32242 (3140–69643)	51	25	17.16 (8.54–33.68)	21.8 (9–43)	1.65 (0.85–2.76)	4.31 (1.52–10.02)

We used Random Forests [RF; 50] to determine if any bacterial taxa were predictive of classifying a sample to vaccination state using the Rvax-start, Rvax, and Rvax-infect as subgroups. We first identified the optimal number of features (OTUs) to include in constructing regression trees to be that with the median out-of-bag (OOB) error rate at the asymptote. We used the parameter ntree = 1000 for the total number of trees to grow in the forest and set the parameter importance to TRUE to assess importance of the different features in prediction and ran five iterations to evaluate consistency of assignment error.

## Results

### Depth, OTUs, Genera, and Alpha diversity

**Fig 1:**
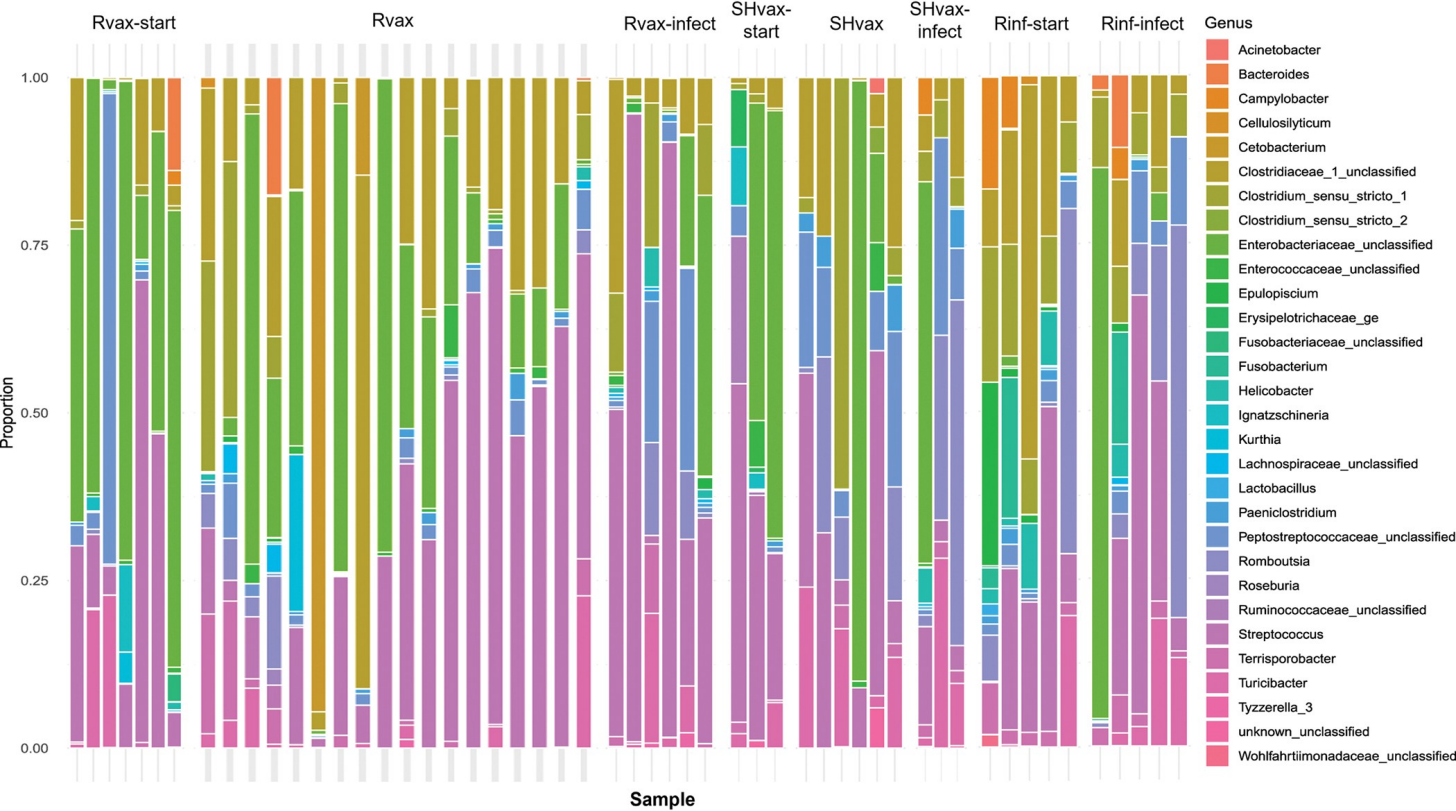
Bar plots of bacterial genera identified from 16S DNA sequences from striped skunk fecal samples. Each color represents the proportion of DNA sequences associated with each genus. Each individual bar plot represents a sample datapoint in each of the treatment subgroups. See Table 1, S1 Table, and methods for description of abbreviated treatment subgroups listed on top of plots.

**Fig 2:**
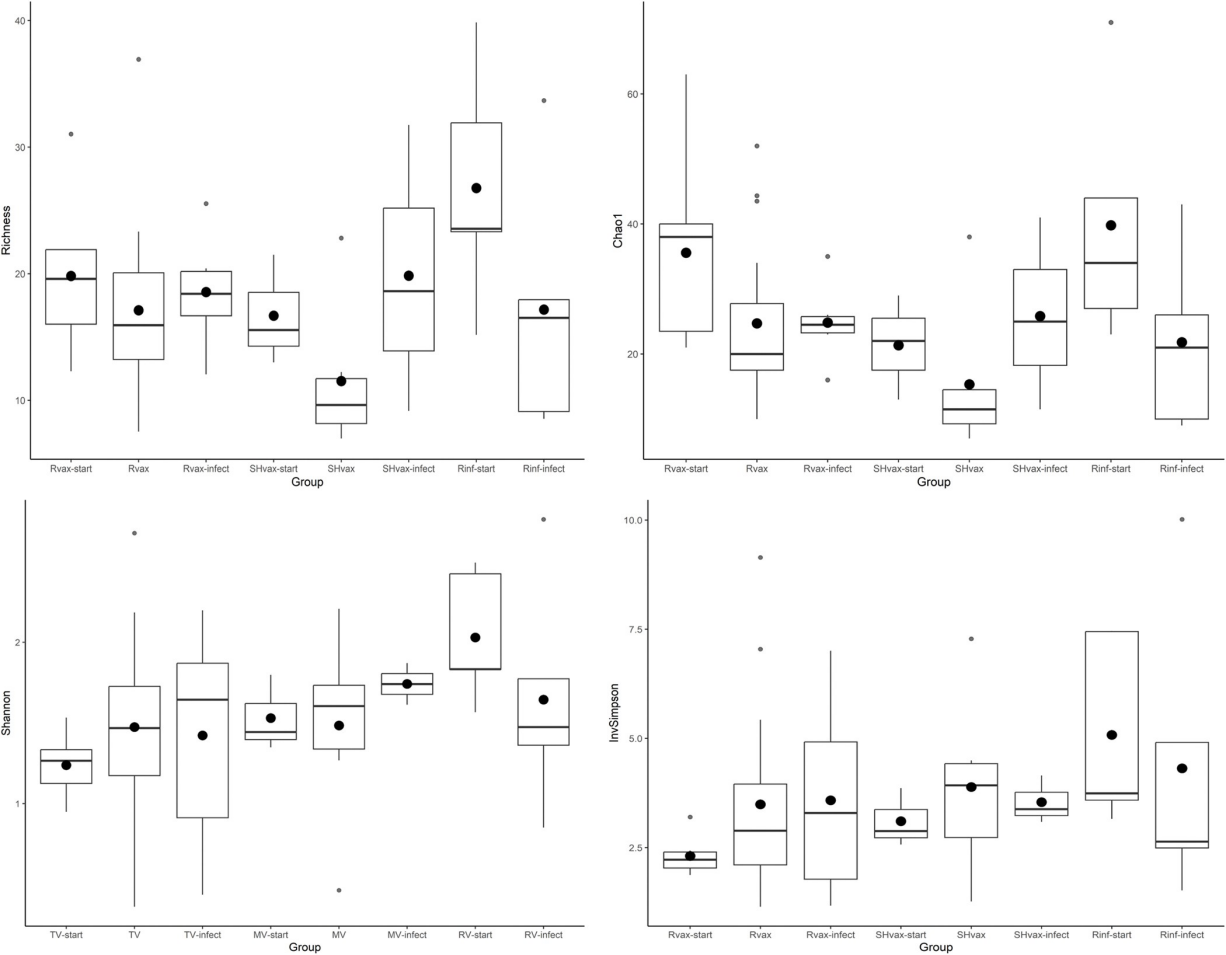
Box plots of alpha diversity metrics estimated from bacterial operational taxonomic units (OTUs) identified from 16S DNA sequences from striped skunk fecal samples. Each plot displays the median as horizontal bar and the mean as a black dot. The metric displayed in each plot is listed on the y-axis and the treatment subgroups is on the x-axis. See Table 1, S1 Table, and methods for definition of treatment subgroups.

Processing, quality filtering, and removal of samples with fewer than 1000 reads, based on rarefaction curves (S1 Fig), resulted in a total of 53 data points obtained through repeated measurements of 15 animals (seven vaccine (Rvax), three sham vaccine (SHvax), and 5 rabies infection only (Rinf; Table 1)). This resulted in 2,818,526 sequence reads with a mean depth per sample of 53,180 reads (range: 1013–168,770; S2 Fig) and 213 operational taxonomic units (OTUs) representing 75 bacterial genera. Raw sequencing read data are available at the National Center for Biotechnology Information’s (NCBI) Sequence Read Archive (SRA) under accession number PRJNA875681. The relative abundances of each bacterial genera are presented Fig 1. The results of the tests for normality tests for sequencing depth, number of datapoints, number of OTUS, and number of genera are presented in S3 Table, with mean depth and number of datapoints showing significant departures. Sequencing depth, number of OTUs and genera, and values of alpha diversity indices for each treatment group are presented in Table 1 and with mean and median plotted in Fig 2 (depth and alpha diversity for each datapoint is provided in S2 Table with OTUs, and genera in S4 and S5 Tables). Only Shannon diversity conformed to a normal distribution based in the Shapiro-Wilks test (*W* = 0.98, *p* = 0.56); Chao1 (*W* = 0.92, *p* = 0.0013), richness (*W* = 0.93, *p* = 0.0053), and inverse Simpson (*W* = 0.86, *p* = 2.2e-05) were not normally distributed. No discernible pattern to the alpha diversity among treatment subgroups is evident from Fig 2 and none of the statistical tests for differences among mean diversities were significant: Chao1 (Kruskal-wallis; *χ*^*2*^ = 13.31, *p* = 0.065), richness (Kruskal-wallis; *χ*^*2*^ = 12.06, *p* = 0.099), inverse Simpson (Kruskal-wallis; *χ*^*2*^ = 8.07, *p* = 0.33), and Shannon (ANOVA; *F* = 1.14, *p* = 0.36). We also tested for differences in alpha diversity based on time of sampling and sex of animal and none of the tests were significant. There were five bacterial phlya present across all samples and the dominant phyla in the skunk microbiome were Firmicutes (61% of OTUs) followed by Proteobacteria (15%). The Firmicutes OTUs were represented predominantly by families Clostridiaceae (26%) followed by Lachnospiraceae (16%), Peptostreptococcaceae (14%), Fusobacteriaceae (12%), with other OTUs comprising less than 10% of the total (S2 Table).

### Beta diversity

**Fig 3:**
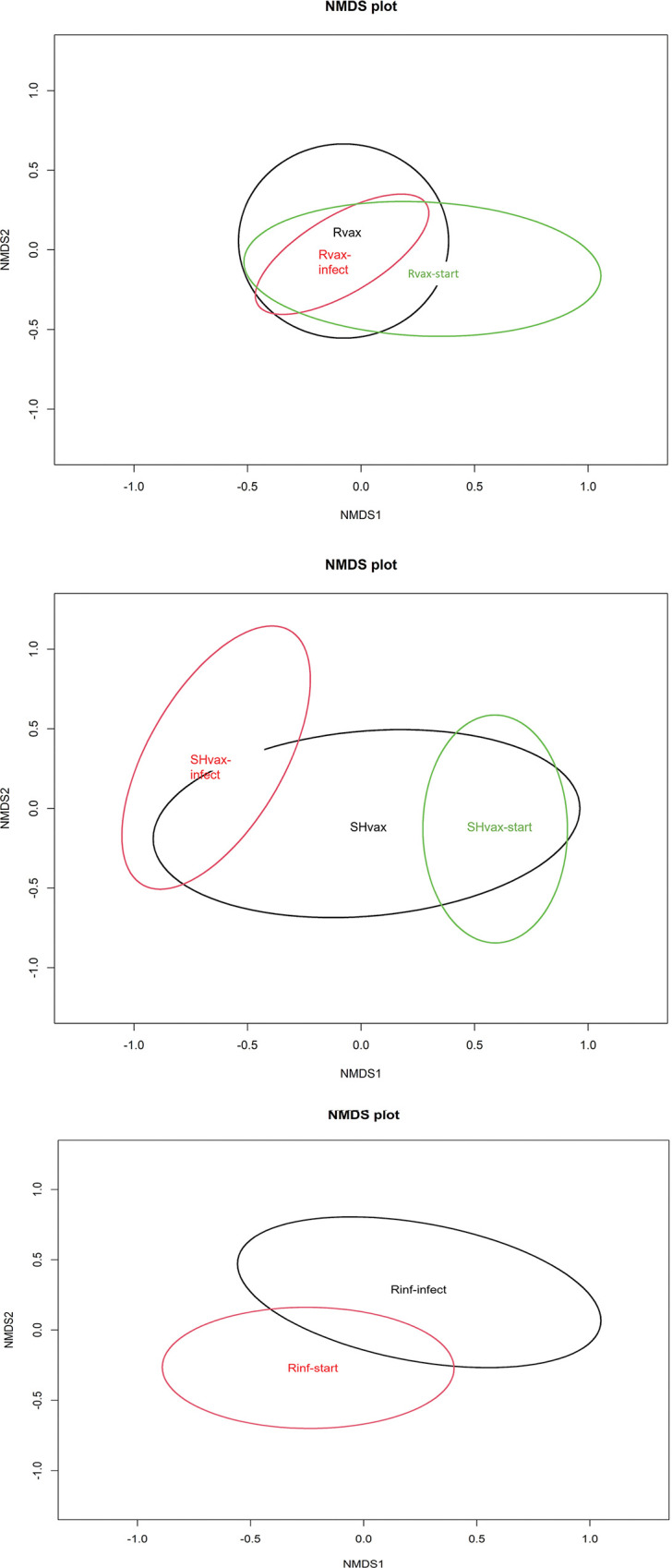
NMDS ordination plots for each treatment group. Individual plots were estimated from Bray-Curtis distance between treatment subgroups. Each subgroup ID represents the group centroid of the 95% confidence ellipsoid.

**Fig 4:**
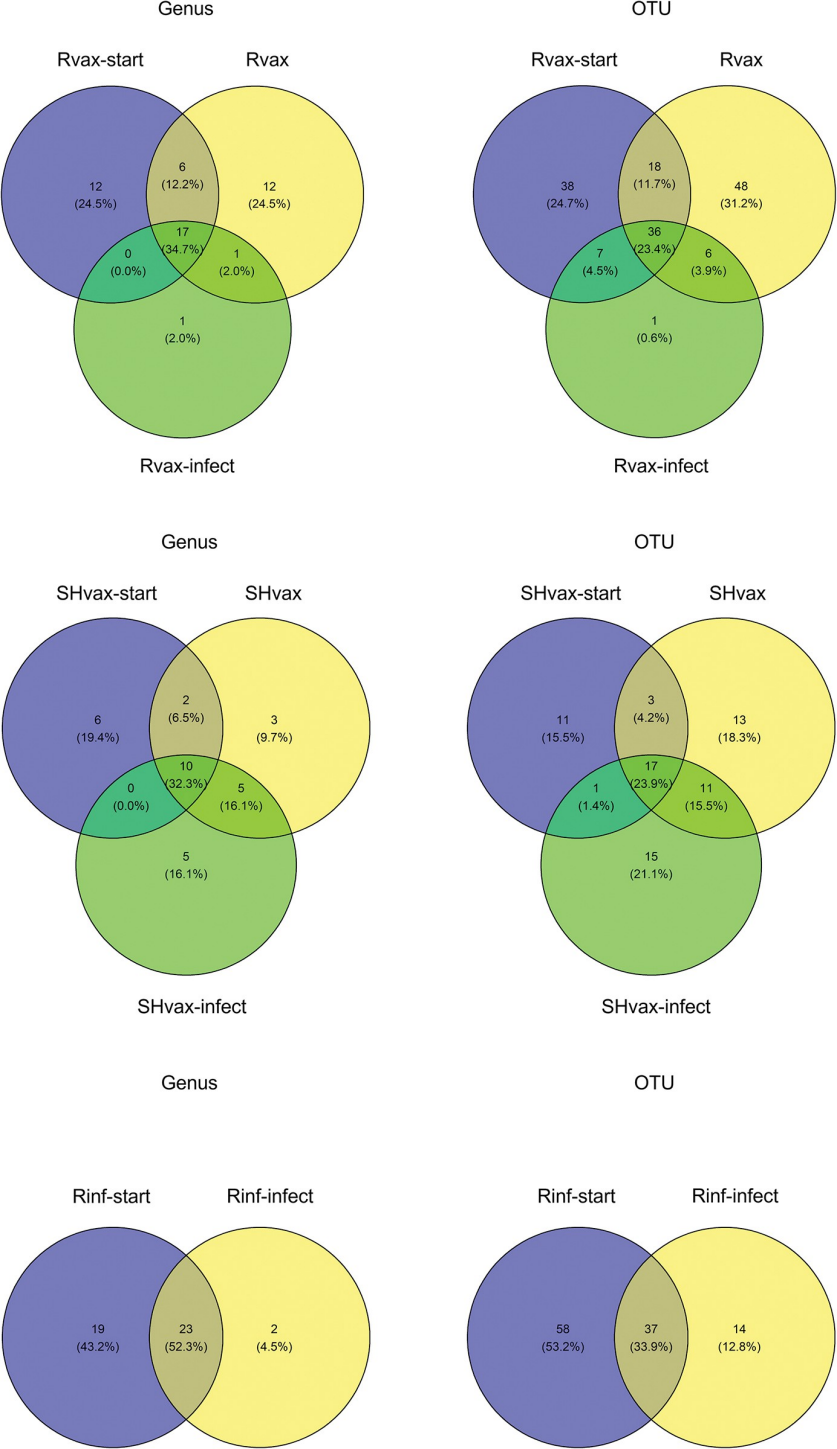
Venn diagrams displaying the shared and unique bacterial operational taxonomic units (OTUs) and genera among treatment subgroups. Each individual plot represents a treatment group. See Table 1, S2 Table, and methods for definition of subgroup abbreviations.

The NMDS plots demonstrate a shift in microbiome diversity among the subgroups within each treatment, Rvax, SHvax, Rinf but each subgroup still showed overlap (Fig 3). However, any structure must be interpreted within the context that only the Rinf plot had stress level below 0.1. But review of the plots demonstrates a shift in microbial communities following rabies virus infection, except in the Rvax group (Fig 3). The PERMANOVA on Bray-Curtis dissimilarity between treatment subgroups was significant for the Rvax group (*F* = 2.11, *p* = 0.009) but not the SHvax (*F* = 0.95, *p* = 0.49) or Rinf group (*F* = 1.56, *p* = 0.11). The shared OTUs among subgroups within each treatment are shown in the Venn diagrams in Fig 4. Notably, the Rvax-infect subgroup only has one unique taxon compared to Rvax-start and Rvax whereas the SHvaxgroup had approximately equal percentages of unique taxa in each subgroup and the Rinf group had a reduction in unique taxa when comparing the Rinf-start and Rinf-infect subgroups. The Rvax group differed from the SHvax group in that the percentage of unique OTUs in the Rvax group was much larger but the taxonomic uniqueness of the Rvax-infect was decreased compared to the SHvax-infect. This unbalance could be due seven sampled animals in the Rvax group versus three animals in the SHvax group.

### Random forests

**Fig 5:**
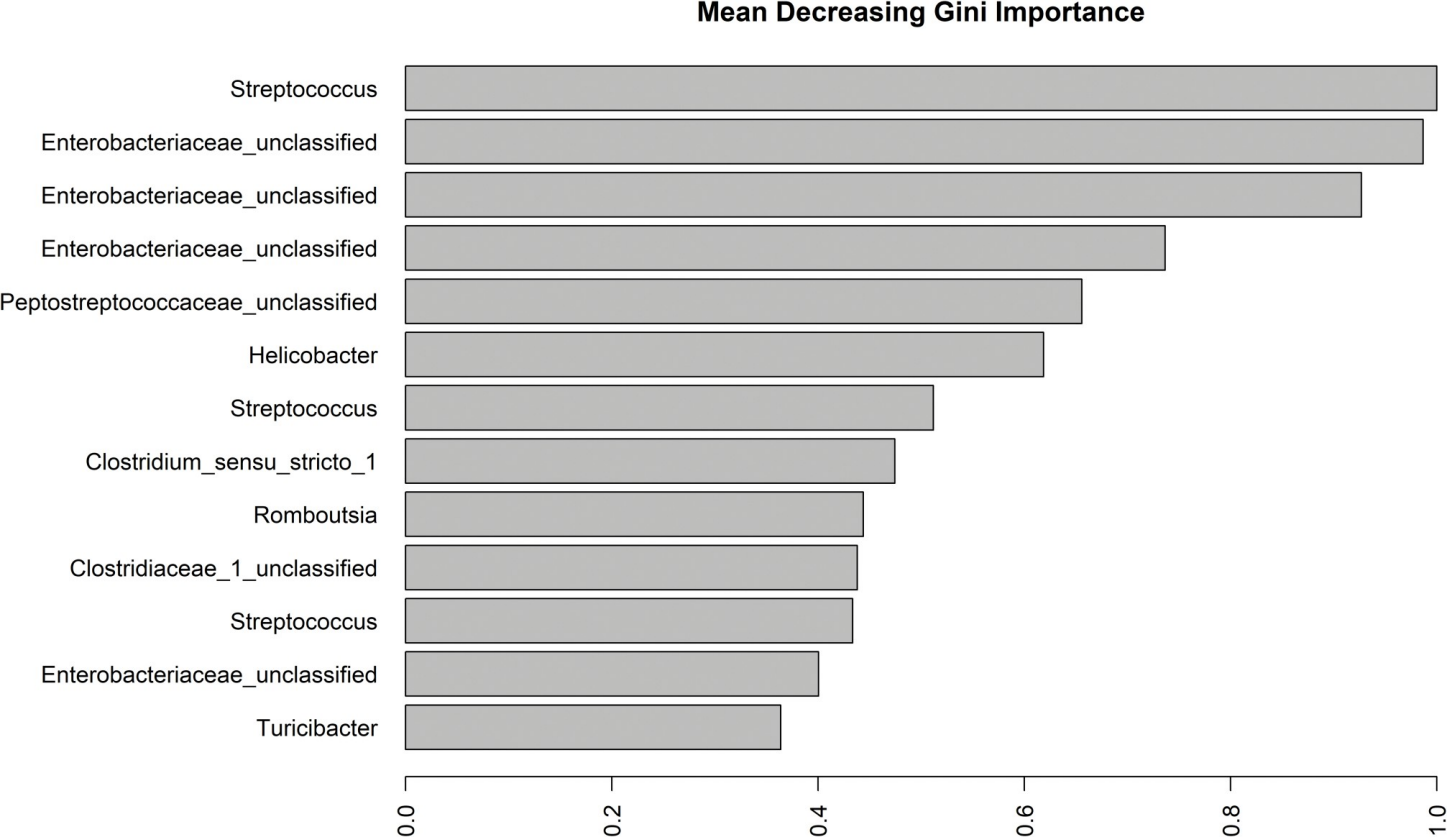
The mean decreasing Gini importance OTU importance estimated with random forests and bacterial 16S DNA sequences from striped skunk fecal samples. The Gini importance measures on the x-axis represent important OTUs for classifying samples in the vaccine (Rvax) treatment group into subgroups. The y-axis is the OTU and associated taxonomy.

Using RF we attempted to identify bacterial OTUs that were important for classifying the samples in the Rvax group into their respective subgroups, Rvax-start, Rvax, and Rvax-infect. Through RF tuning, the median OOB error asymptote was at 44 OTUs with a value of 0.065 (S3 Fig). The mean OOB error rate across 5 runs using 44 OTUs and 1000 trees was 7.1%, which means that approximately 93% of the time samples were assigned to their correct subgroup. The confusion matrix classification error rate for assigning sample to Rvax and Rvax-infect was zero but the mean error rate across the five runs for Rvax-start was 0.34 (S4 Fig). Four to five of the seven Rvax-start samples consistently assigned to the appropriate subgroup. Across four of the RF replicates, two to three of the Rvax-start assigned to Rvax-infect. In a single run, two assigned to Rvax-start and one assigned to Rvax. Fig 5 and S6 Table show the 13 OTUs with the largest influence on cluster assignment. Of these 13, two belong to the family Clostridiaceae, three belong to the Streptococcaceae (specifically the genus *Streptococcus*), four belong to the family Enterobacteraciae, one belongs to the Erysipelotrichaceae (specifically the genus *Turcibacter*), two in the Peptostreptococcaceae family, with a single OTU each in the Helicobacteraceae (genus *Helicobacter*). The important OTUs belong to four orders in three phyla, Proteobacteria, Firmicutes, and Epsilonbacteraeota.

### Comparison to VOC data

**Fig 6:**
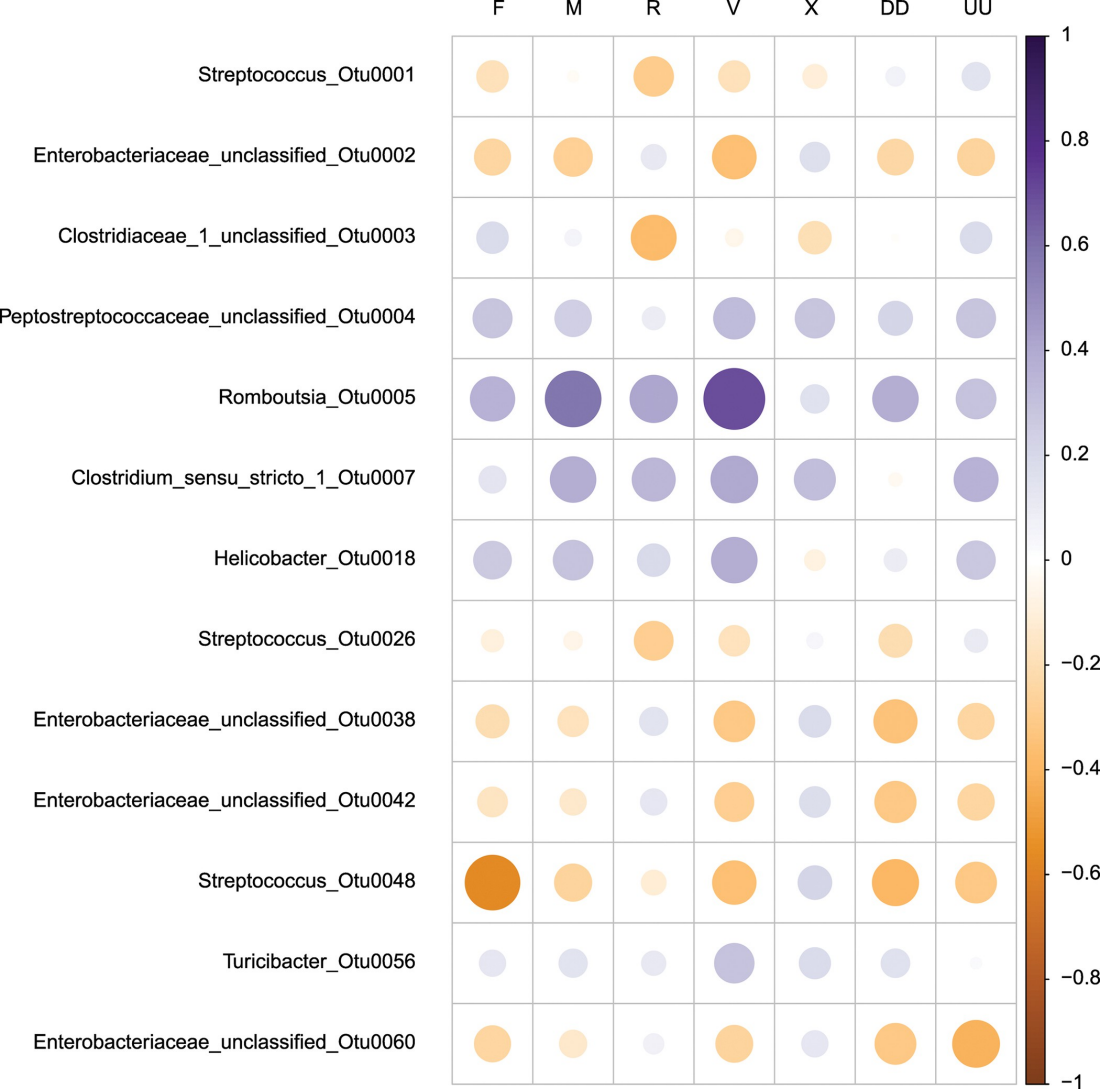
Correlation plot between bacterial operational taxonomic units (OTUs) and volatile organic compounds (VOCs) collected from striped skunk fecal samples. The size increases and color darkens with increasing or decreasing Spearman rank correlation coefficient. The codes for the VOCs are in S5 Table and values for correlation coefficients are in Table 2. Each of the OTUs was identified in this study as important for classifying samples into vaccination treatment (Rvax) subgroups (see Table 1). The VOCs were identified by Kimball et al., (2019) as correlated with striped skunk vaccination status.

Spearman rank correlation results for 13 OTUs with the largest mean Gini importance compared to seven VOCs that had a significant positive correlation with vaccination status in skunks from Kimball, Volker [12] are shown in Fig 6 and Table 2. The largest positive correlation was 0.70 and the lowest was -0.57. There were two correlations greater than 0.50 and one less than -0.50. There were 23 instances of p-values less than 0.1 and 14 instances of values less than 0.05 (without correction for multiple comparisons). After correction for multiple comparisons using false discovery rate there were only three significant tests less than 0.1 and all of these were less than 0.05. Two of the significant tests, which were associated with positive correlations, were from comparisons with the *Romboustia* genus (ninth highest Gini importance of 0.44) and two different VOCs, 2-pentylfuran and unknown compound A. *Romboustia* also had three more tests that were significant with other VOCs but these were without correction for multiple testing. The remaining significant test was for associations with a *Streptococcus* OTU and it was a negative correlation coefficient. The unknown compound A was the VOC that had the most correlations with p-values less than 0.1 without correction, and one that was significant following correction.

**Table 2:** Spearmen rank correlation coefficients from comparison of bacterial OTUs and volatile organic compounds (VOC) collected from stripe skunk fecal samples. Each of the OTUs was identified in this study as important for classifying samples into vaccination treatment (Rvax) subgroups (see Table 1). The VOCs were identified by Kimball et al,. (2019) as correlated with striped skunk vaccination status and the letters in parentheses are the VOC codes from Kimball et al., 2019. Values in bold were statistically significant (p < 0.10) without correction, * are values that were signficant followig false discovery rate correction, and correlation coefficients over 0.5 and under -0.5 are underlined.

Taxa_OTU	Hexanal (F)	2-pentylfuran (M)	4-nonanone (R)	Unknown compound A (V)	1-octen-3-ol (X)	Benzaldehyde (DD)	2-methyl quinoline (UU)
Streptococcus_Otu0001	-0.19	-0.02	-0.29	-0.18	-0.11	0.07	0.15
Enterobacteriaceae_unclassified_Otu0002	-0.24	-0.28	0.12	**-0.36**	0.16	-0.24	-0.25
Clostridiaceae_1_unclassified_Otu0003	0.19	0.05	**-0.38**	-0.06	-0.20	-0.01	0.19
Peptostreptococcaceae_unclassified_Otu0004	0.29	0.25	0.10	**0.32**	0.29	0.22	0.28
Romboutsia_Otu0005	**0.37**	0.58*	**0.42**	0.70*	0.15	**0.39**	0.30
Clostridium_sensu_stricto_1_Otu0007	0.14	**0.39**	**0.35**	**0.41**	**0.32**	-0.03	**0.36**
Helicobacter_Otu0018	0.27	0.30	0.20	**0.39**	-0.08	0.10	0.28
Streptococcus_Otu0026	-0.09	-0.06	-0.28	-0.17	0.05	-0.20	0.10
Enterobacteriaceae_unclassified_Otu0038	-0.20	-0.17	0.15	**-0.31**	0.19	**-0.35**	-0.24
Enterobacteriaceae_unclassified_Otu0042	-0.17	-0.13	0.13	-0.28	0.18	**-0.32**	-0.25
Streptococcus_Otu0048	-0.56*	-0.26	-0.11	**-0.35**	0.21	**-0.40**	**-0.31**
Turicibacter_Otu0056	0.13	0.15	0.11	0.29	0.18	0.15	0.02
Enterobacteriaceae_unclassified_Otu0060	-0.24	-0.14	0.08	-0.26	0.13	**-0.31**	**-0.41**

## Discussion

The goal of this study was to evaluate whether oral rabies vaccination alters the skunk fecal microbiome and if these changes can be used to predict an animal’s vaccination status. Using samples collected from individuals that were given an oral vaccine, a sham vaccine, and/or infected with rabies virus, we evaluated the alpha and beta diversities, used Random Forests to identify taxa that are indicative of vaccination status, and compared these data to fecal VOC data from Kimball, Volker [12] collected from the same samples. We determined that alpha diversity was not different among the subgroups within the vaccination, sham vaccine, or rabies virus infection treatments. The NMDS plot demonstrated that no major shifts among groups were visible but there were trends. However, the beta diversity was significant between subgroups within the vaccine group (Rvax), but not between subgroups in the sham vaccine or rabies virus infection only groups. Furthermore, there were bacterial taxa that had significant impact on classifying samples into their respective treatment subgroups within the rabies vaccination group. Finally, there were positive correlations between OTUs and VOCs that demonstrated correlation with vaccination status.

Our hypothesis was rooted in previous work that demonstrated microbiomes respond to both vaccination and pathogen infection [e.g., 7, 8, 51]. Counter to these previous studies, alpha diversity did not differ among treatment subgroups. Yet, the beta diversity among the vaccine treatment subgroups was statistically significant. Given that beta diversity was significantly different, and RF identified bacterial taxa that classified of samples into vaccination subgroups, suggest that community composition shifted in response to vaccination. If beta diversity is the only metric that reveals vaccine ingestion, then before and after samples would be required which could limit this method. However, to test the replicability of these results and mechanisms for differences in beta diversity, but not alpha diversity, a more expansive study is needed.

In this study, we documented biodiversity of striped skunk fecal microbiome from a rabies vaccine efficacy trial. The two dominant bacterial phyla identified in our samples were Firmicutes and Proteobacteria. These two phyla have also been identified as prominent in most of the mammalian gastrointestinal tract microbiome studies to date, and specifically, Firmicutes is the dominant phylum in carnivore gut microbiomes [52]. Mammalian gut microbiota are known to be labile and shift in response to changes in diet [53]. For this study, each individual animal was captive raised and fed an identical diet during the experiment and we expect that captive skunk microbiomes may have differences compared to wild skunks, as observed for other mammals [54]. Captive mammals with exposure to humans often demonstrate a “humanization” of the gut microbiome which would impact generalizing inferences from studies on captive animals to wild ones [55]. One aspect that validates the use of captive animals for basic mechanistic responses of microbiome to immunological or physiological changes is that host phylogeny dictates community composition, but not OTU abundance, and mammals may demonstrate the strongest association of phylogeny and microbiota among vertebrate clades [2]. Thus, we can hypothesize that captive skunks would have a core microbiome with broad patterns of community composition and responses similar to wild skunks, however this must be empirically tested before extrapolation. A complete understanding of the effects of phylogeny, life stage, habitat, season, physiological state (e.g., gestation, torpor), and immunological status is critical for validation of a reliable microbiome biomarker in wild animals.

Some, but not all, of the bacteria taxa with the greatest influence on sample classification were correlated with VOCs and/or have been associated with oral vaccination. *Streptococcus* was the most influential OTU on RF classification which corresponds to previously identified association of gut *Streptococcus* to inflammation in domestic dogs and cats [56, 57]. Of the highly influential OTUs on RF classification the Enterobacteriaceae were represented among the largest Gini importance factors but were negatively, or slightly positively, correlated with the VOCs hexanal (F), 2-pentylfuran (M), 4-nonanone (R), and unknown compound A (V) of which 2-pentylfuran is a known bacterial metabolite [58]. The Enterobacteriaceae include a wide range of species associated with gut microbiota in mammals with diverse ecological niches. Since these OTUs belong to unclassified genus/genera and are unknown species, little can be said about the role these may play in the response to vaccination and/or infection. Both Abdo, LeCureux [7] and Zhang, Wu [6] found that the order Clostridiales (families Clostridiaceae, Lachnospiraceae (genus *Tyzzerella*) and Peptostreptococcaceae (genus *Romboustia* from current study) were associated with vaccination classification and immune response. Specifically, Zhang, Wu [6] identified that abundance of unclassified Clostridiales and Lachnospiraceae OTUs were positively correlated with rabies vaccine immune response in mice.

We found *Romboustia* had the strongest correlation with VOCs and other studies corroborate our results that this genus is associated with metabolites dependent on disease and study model, and with dysbiosis in the microbiome and host inflammation. In a murine *ApoE*^*-*^*/*^*-*^ model for atherosclerosis, Yan, Wang [59] determined metformin treatment altered microbiome diversity in a treatment-dependent manner; specifically, *Romboutsia* decreased in abundance due to metformin treatment and was positively associated with inflammatory immune markers and negatively correlated with various short chain fatty acids. Jin, Jia [60] also found *Romboutsia* was positively correlated with TNFα and the metabolites methoxy-4-hydroxypheylglycol sulfate and cis-(6,9,12)-Linoleic acid in Bamei suckling piglets given a milk replacer supplement, and negatively correlated with genes regulating intestinal barrier permeability and function (including ZO-1) however association with specific immune functions has not been determined.

Logical next steps to improve and expand this study would be to increase treatment group sample sizes, evaluate wild-caught striped skunks versus captive raised, test other rabies reservoir species and targets of management such as raccoons, and determine if the ingestion of the oral bait to deliver the rabies vaccine versus direct instillation has similar effects on the host microbiome response. The oral rabies vaccine bait used for raccoon and canid rabies management in the U.S. uses tetracycline as a biomarker. Detecting the biomarker is invasive as it requires anesthetizing an animal and removing a tooth [29]. Identifying a fecal biomarker of vaccine bait uptake would improve the ease of sample collection for population monitoring and reduce the risk to field biologists and animal welfare, but characterization of the limits to sample freshness, number of samples, and regularity of collection would all need to be investigated. Another interesting test would be to explore the impact of tetracycline in the oral bait on the gut microbiome. Tetracycline is an antibiotic and would likely impact the gut microbiota [61]. Perhaps in carnivores the ingestion of tetracycline can leave a signature in the fecal microbiome that would allow for detection or recent bait ingestion. The oral vaccine used in this study did not contain tetracycline but the MEM culture media used to dilute the vaccine and prepare sham treatments did contain low levels of an antibiotic and antimycotic. But our study design accounted for this by incorporating the sham vaccine to test for artifacts of either animal handling or the vaccine media. We saw different responses in the vaccinated versus sham groups which suggests that the MEM media was not a major factor the experimental outcomes.

Biomarkers have high potential to facilitate non-invasive monitoring of populations for pathogens and vaccination status, which is critical to wildlife disease management. Continually pushing the boundaries of sample types and diagnostic molecules will lead to increased efficiency, decreased cost and risk, and open new doors to additional types of diagnostic samples for wildlife disease surveillance. In this study we identified some correlations between fecal bacterial taxa, vaccination status, and VOCs. While we cannot infer beyond the results to identify causal mechanisms, these results can lead to additional hypotheses and further studies about new biomarkers to assist with rabies, and other pathogen surveillance in wildlife.

## Supporting information

S1 Table Indexes. Indexes used for each sample in this study of skunk microbiome.
(XLSX)

S2 Table Sample metadata and diversity metrics. Summary data for each sample datapoint (ID) collected for the skunk microbiome study. Includes are Animal ID, animal sex, date of collection, dose of rabies virus infection, the treatment group associates with data analysis (Table 1), treatment subgroup, days post vaccination, days post infection, rabies virus neutralizing antibody (rVNA) from Kimball et al., 2019, and alpha diversity metrics based on OTUS for each individual sampling datapoint.
(XLSX)

S3 Table Shapiro-Wilks test. Results from Shapiro-Wilks test for normality of the data across all samples.
(XLSX)

S4 Table OTUs and taxonomy. Operational taxonomic units (OTU) and associated taxonomy of 16S bacterial sequences collected from striped skunk fecal samples.
(XLSX)

S5 Table Sequencing depth. Operational taxonomic units (OTUs) of bacterial 16S DNA sequences collected from striped skunk fecal samples and sequence depth for each sample data point. See S1 Table for treatment groups associated with datapoints.
(XLSX)

S6 Table Random Forests OUT importance. Operational taxonomic units (OTUs) and associated taxonomy identified from bacterial 16S DNA sequences collected from striped skunk fecal samples. The relative mean Gini importance (a) estimated by random forests for 44 OTUs and 1000 trees that influence the classification of TV datapoints into treatment subgroups: TV-start, TV, and TV-infect.
(XLSX)

S7 Table VOCs from Kimball et al. (2019). Volatile organic compounds (VOC) identified in striped skunk fecal samples and their code used in Fig 6. Table was adapted from Kimball et al., (2019).
(XLSX)

S1 Fig Rarefaction curves based on operational taxonomic units from skunk fecal microbiome. (PDF)

S2 Fig Histogram of sequencing depth per sample for skunk fecal microbiome samples. (PDF)

S3 Fig Random Forests Tuning results for skunk fecal microbometaxa based on 1000 trees. X-axis Represents the number of OTUS included in the permutation and y-axis is the median out of bag error.
(PDF)

S4 Fig Random Forests classification error rate for skunk fecal microbiome samples representing vaccinated (TV), Before any treatment (TV-start), and post-vaccinrabies virus challenge (TV-infect). The lines represent the out of bag (OOB) error rate (y-axis) for the number of trees included in the analysis (x-axis).
(PDF)
