# Supplementary figures and images for: Biodiversity indices and Random Forests reveal the potential for striped skunk (Mephitis mephitis) fecal microbial communities to function as a biomarker for oral rabies vaccination
Source: PLoS One. 2023 Aug 22;18(8):e0285852. doi: 10.1371/journal.pone.0285852 (PMC10443867; doi:10.1371/journal.pone.0285852)

S1 Fig. Rarefaction curves based on operational taxonomic units from skunk fecal microbiome.

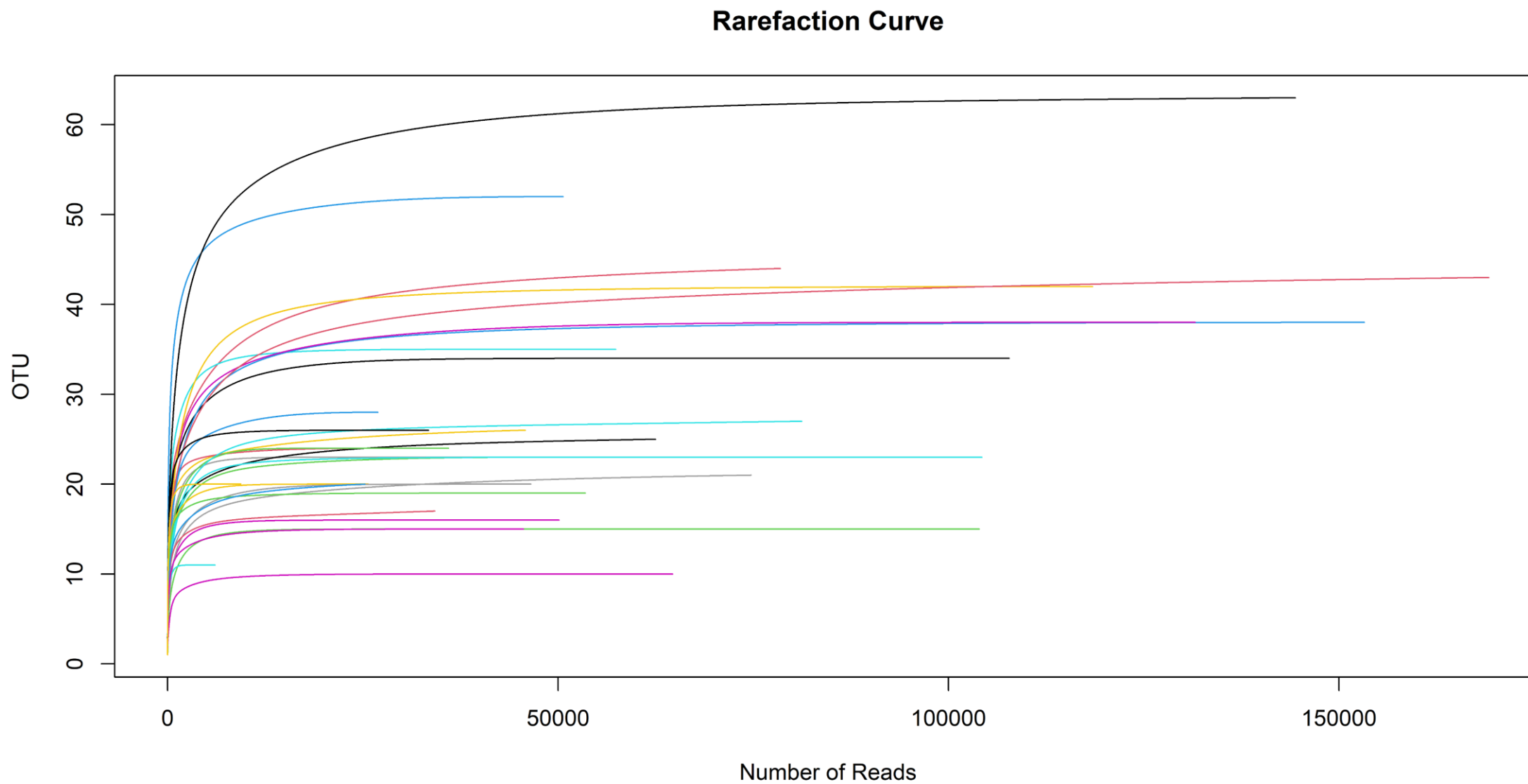

Supplement: S1 Fig — (PDF) [file pone.0285852.s008.pdf]

S2 Fig. Histogram of sequencing depth per sample for skunk fecal microbiome samples.

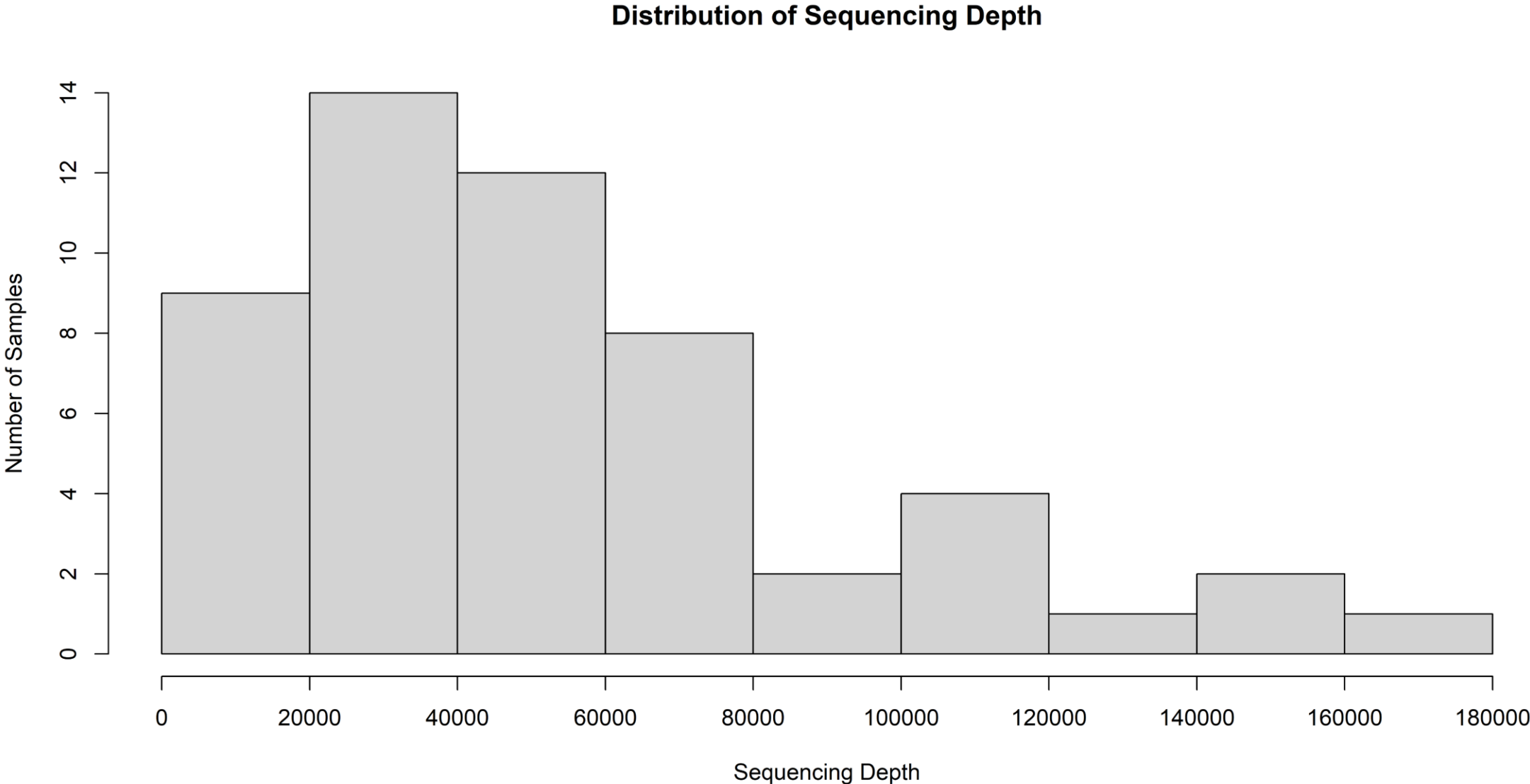

Supplement: S2 Fig — (PDF) [file pone.0285852.s009.pdf]
