# Supplementary material for: Biodiversity indices and Random Forests reveal the potential for striped skunk (Mephitis mephitis) fecal microbial communities to function as a biomarker for oral rabies vaccination
Source: PLoS One. 2023 Aug 22;18(8):e0285852. doi: 10.1371/journal.pone.0285852 (PMC10443867; doi:10.1371/journal.pone.0285852)

S3 Fig. Random Forests Tuning results for skunk fecal microbome taxa based on 1000 trees. X-axis Represents the number of OTUS included in the permutation and y-axis is the median out of bag error.

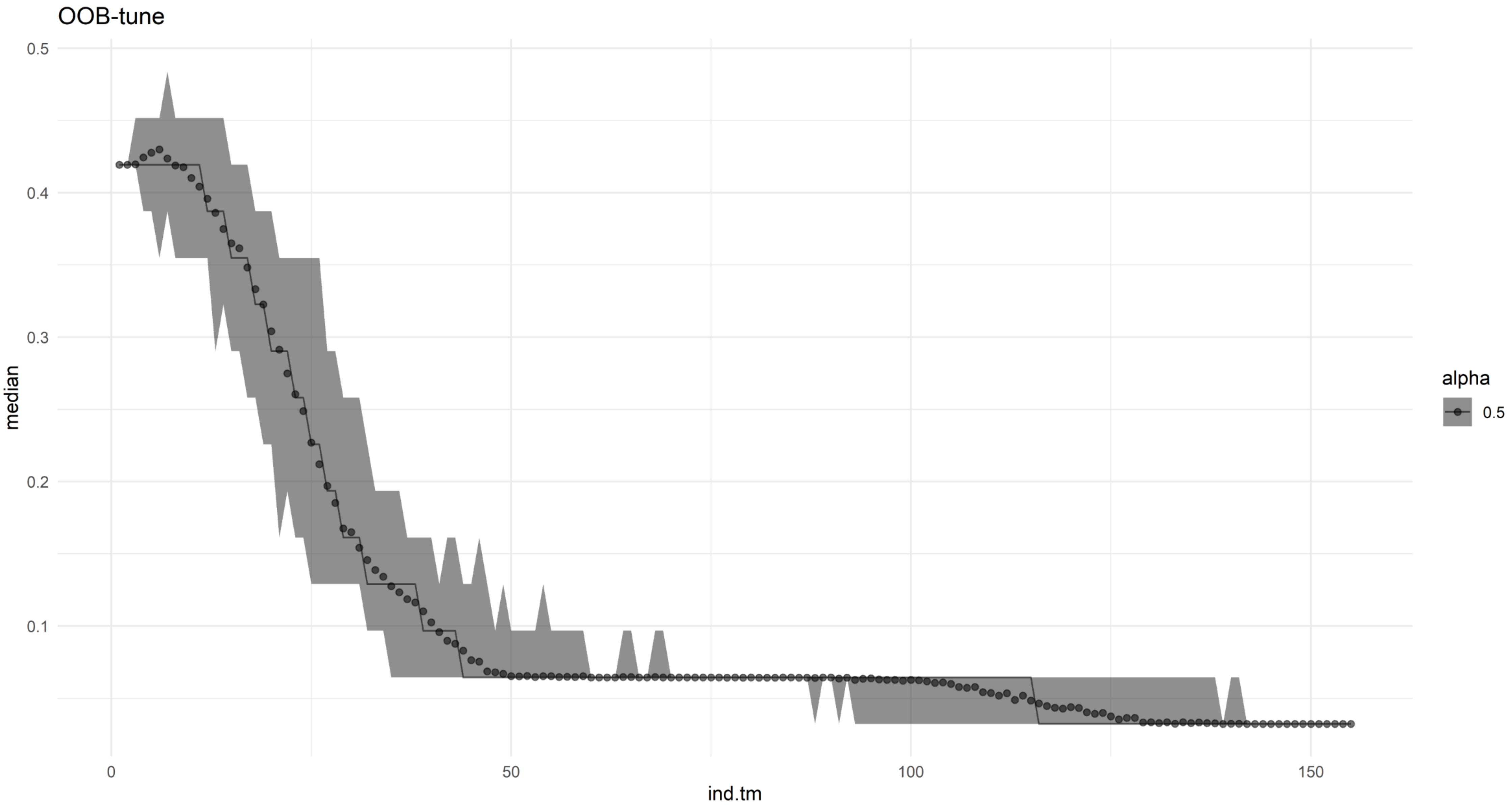

Supplement: S3 Fig — X-axis Represents the number of OTUS included in the permutation and y-axis is the median out of bag error. (PDF) [file pone.0285852.s010.pdf]
