# Supplementary material for: Biodiversity indices and Random Forests reveal the potential for striped skunk (Mephitis mephitis) fecal microbial communities to function as a biomarker for oral rabies vaccination
Source: PLoS One. 2023 Aug 22;18(8):e0285852. doi: 10.1371/journal.pone.0285852 (PMC10443867; doi:10.1371/journal.pone.0285852)

S4 Fig. Random Forests classification error rate for skunk fecal microbiome samples representing vaccinated (TV), Before any treatment (TV-start), and post-vaccin rabies virus challenge (TV-infect). The lines represent the out of bag (OOB) error rate (y-axis) for the number of trees included in the analysis (x-axis).

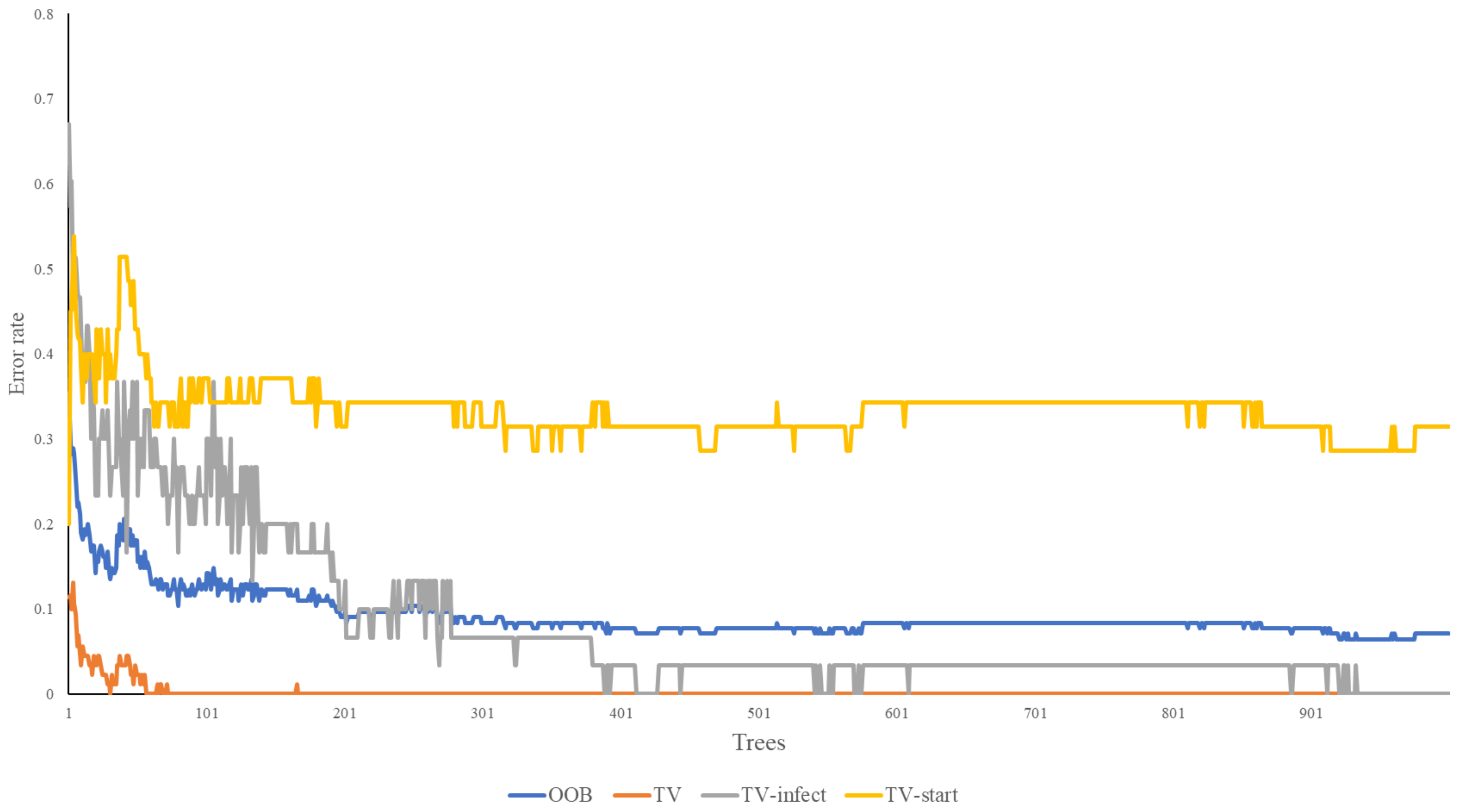

Supplement: S4 Fig — The lines represent the out of bag (OOB) error rate (y-axis) for the number of trees included in the analysis (x-axis). (PDF) [file pone.0285852.s011.pdf]
